# Supplementary material for: Comparative multiomics analysis of cell physiological state after culture in a basket bioreactor
Source: Sci Rep. 2022 Nov 23;12:20161. doi: 10.1038/s41598-022-24687-4 (PMC9686226; doi:10.1038/s41598-022-24687-4)

23666045.1:36606971:36607113:-@NW_023666045.1:36600756:36600887:-@NW_023666045.1:36600502:36600

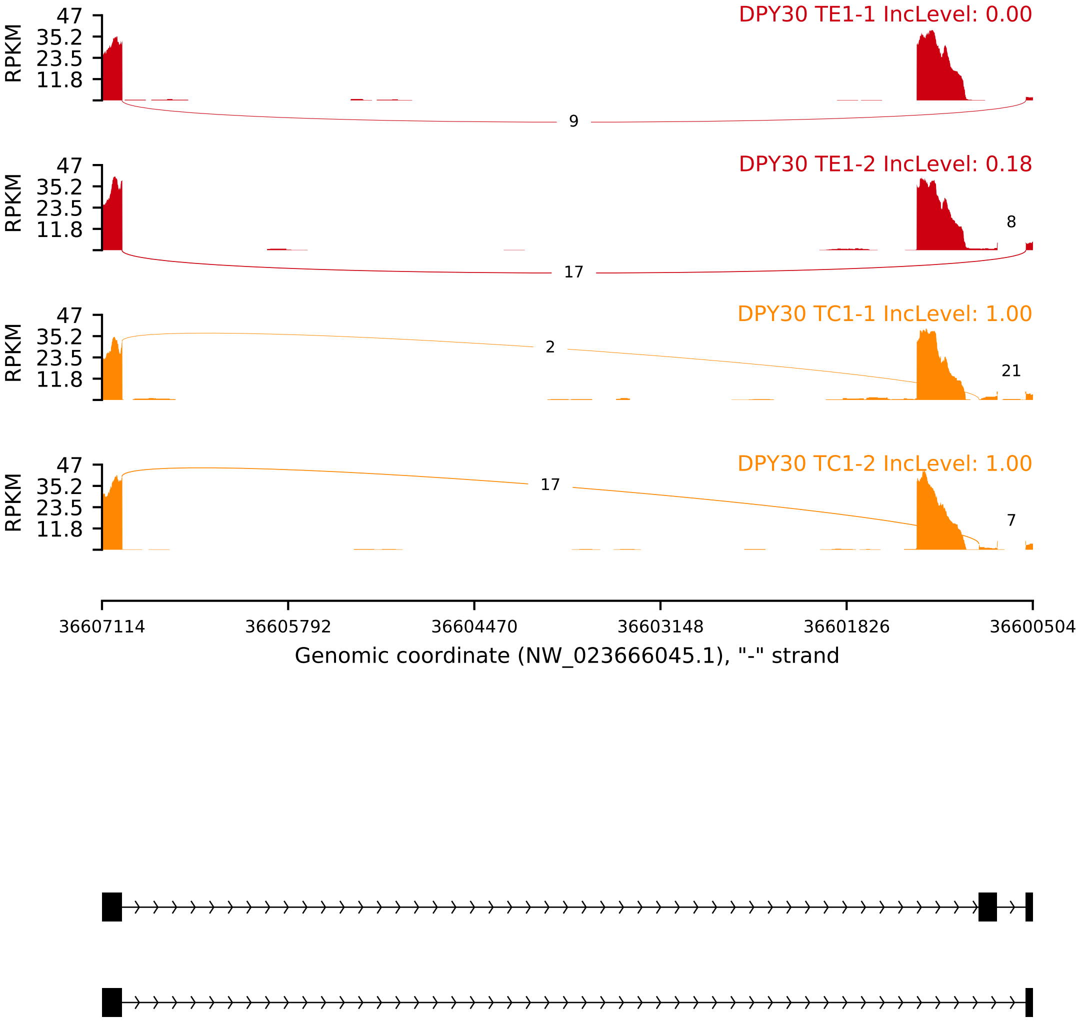

Supplement: Supplementary file 1 — Supplementary Information 1. [file 41598_2022_24687_MOESM1_ESM.zip › raw data/Transcriptomic raw data/7.AS/2.dasplot/TE1vsTC1/SE/1_DPY30_NW_023666045.1_36606971_36607113_-@NW_023666045.1_36600756_36600887_-@NW_023666045.1_36600502_36600554_-.pdf]
